# Does surgical approach affect outcome after fixation of intra-articular fractures of distal humerus? Retrospective cohort study from a level-1 trauma centre in a metropolitan city

**DOI:** 10.1016/j.amsu.2019.05.012

**Published:** 2019-06-03

**Authors:** Mohammad Atif, Obada Hasan, Yasir Mohib, Rizwan Haroon Rashid, Pervaiz Hashmi

**Affiliations:** a Central Park Medical College, Lahore, Pakistan; b Department of Surgery, Section of Orthopedics, The Aga Khan University Hospital, Pakistan

**Keywords:** Intra-articular fractures, Humerus, Osteotomy, Elbow joint

## Abstract

**Introduction:**

Fractures around the distal humerus fractures make up to 2% of all fractures. Complex intra-articular distal humerus fractures present as challenge to restore of painless, stable and mobile elbow joint. Surgical exposure to all critical structures is of paramount importance to achieve anatomic reduction. Conflict still persists regarding the choice of ideal approach. In this study we compare the effect of surgical approach triceps lifting vs olecranon osteotomy on the functional outcome after fixation of distal humerus fractures.

**Methods:**

Non-funded, non-commercial, retrospective cohort study was conducted on patients with closed distal humerus intra-articular fractures between 2010 and 2015 at our tertiary care level-1 trauma and university hospital. Patients >18 years of age with closed complex intra-articular distal humerus fracture were operated using one of the two surgical approaches, either triceps lifting approach (Group1) or with olecranon osteotomy (Group 2). Functional evaluation using quick DASH scores at 1 year of follow-up. Study is registered with ID:NCT03833414 and work has been reported in line with the STROCSS criteria.

**Results:**

Out of 43 patients 16 were treated with triceps lifting approach and 27 with olecranon osteotomy. The difference between the mean quick DASH score for both groups was not statistically significant (p = 0.52) although higher for group 1. Complications were comparable for both groups but 2 patients suffered delayed union of osteotomy site in group 2.

**Conclusion:**

Triceps lifting approach can be used equally efficiently for exposure of these complex distal humerus injuries with no comprise in visibility of articular fragments.

## Highlights

• Complex intra-articular distal humerus fractures present as challenge to restore of painless, stable and mobile elbow joint.
• Surgical exposure to all critical structures is of paramount importance to achieve anatomic reduction.
• For long time, olecranon osteotomy approach was utilized to provide clear access to the joint.
• This is at the cost of creating osteotomy and the risk of its delayed/nonunion.
• Triceps lifting approach avoids this catastrophic complication.

## 1 Introduction

Of all fractures in the body, distal humerus fracture is about 0.5%–2%, 30% of which involving the articular surface [1]. Among these injuries complex intra-articular distal humerus fractures presents as challenge to even the most experienced surgeon [2,3]. Restoration of painless, stable and mobile elbow joint to resume the patient's necessary activities is essential and depends on the anatomic reduction of the intraarticular component of fracture and stable fixation to allow rehabilitation. To achieve this objective, surgical exposure to all critical structures is of paramount importance [4,5]. Out of the various described techniques each has its own merits and demerits, controversy exists regarding the choice of optimal approach [6].

Zhang et al., in 2013 at Shanghai observed reductions in procedure times, blood loss, complication rates and improved outcomes (all *P* < 0.01) with the triceps-sparing approach compared with olecranon osteotomy [7]. Chen et al., in 2010 found no statistically significant difference in functional outcome by using either of these approaches. On review of Literature conflict still persist regarding the choice of ideal approach [8].

In this study, we compared the effect of two surgical approaches (triceps lifting vs. olecranon osteotomy) on the functional outcome after fixation of distal humerus intra-articular fractures. The objective of this study is to compare the difference in functional outcome after fixation of complex distal humerus intra-articular fractures by triceps lifting vs. olecranon osteotomy approach.

## 2 Materials and methods

Non-funded, non-commercial, single centre, retrospective cohort study that was conducted on patients operated for closed distal humerus intra-articular fractures between January 2010 and January 2015 at our tertiary care level-1 trauma and university hospital. Protocol was developed before study start-up and is available from guarantor on request. Study started after obtaining the Ethical Review Committee (ERC) approval of our hospital (3799-Sur-ERC-15). Study is registered at clinicaltrials.gov with ID: NCT03833414. Data were collected, managed and analyzed by the musculoskeletal service line team members including an experienced research associate and trauma consultants. All cases were operated by a single surgeon, who is the senior author of this paper and the musculoskeletal service line chief at our institute with special interest in upper limb reconstructive procedures. The work has been reported in line with the STROCSS criteria [11].

All patients of >18 years of age with closed complex intra-articular distal humerus fracture (Intercondylar Fracture Riseborough Radin Classification type ll and lV) were included. Patients with CVA (Cerebero-Vascular Accident), dementia and associated with neurovascular injuries which may impede with rehabilitation are excluded. Patients were operated using two different surgical approaches either triceps lifting approach (Group 1) or with olecranon osteotomy (Group 2) at the discretion of operating surgeon.

**Fig. 1:**
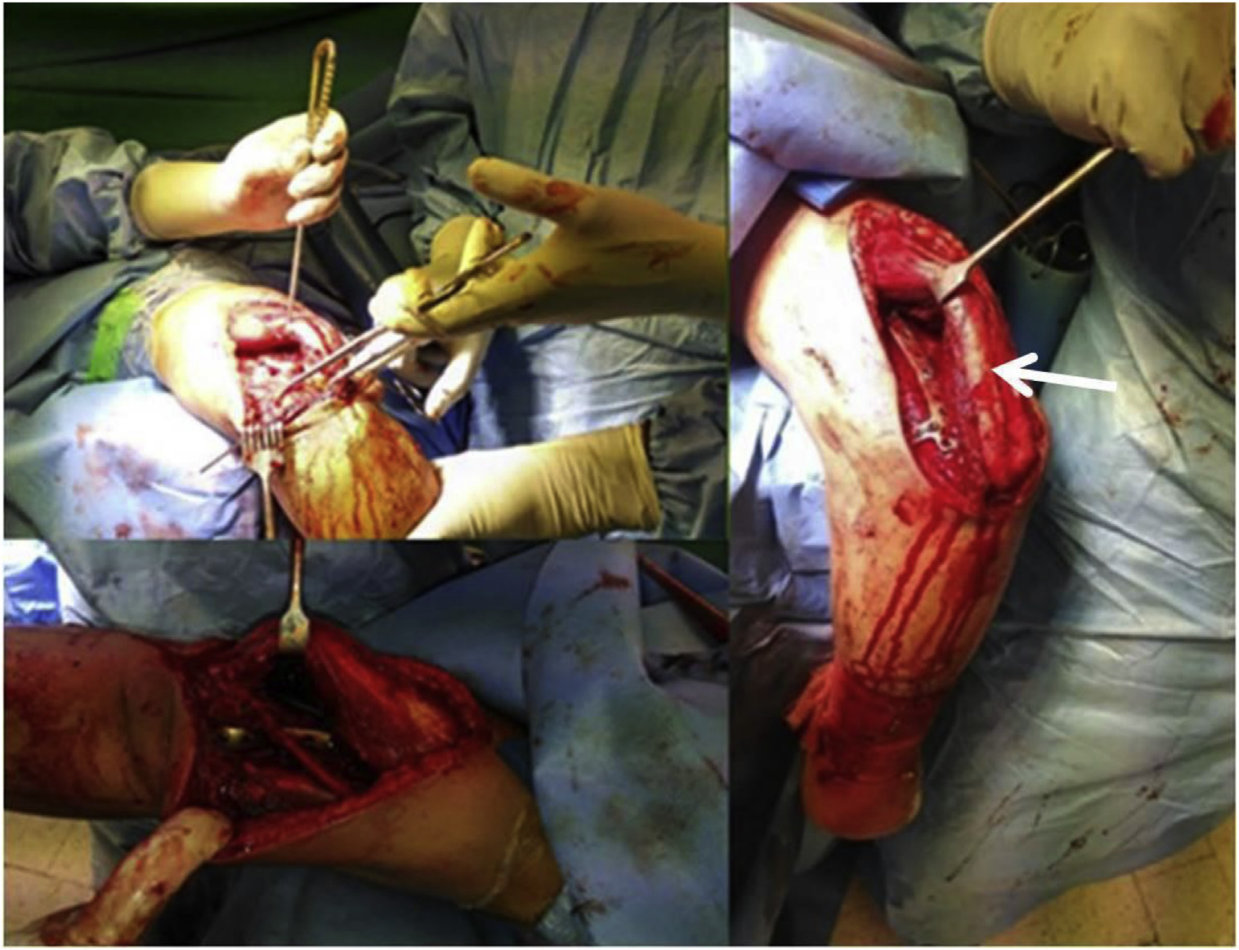
Intraoperative exposure and fracture fixation through triceps lifting approach. White arrow showing the retraction of triceps muscle and the position of plate after fixation without the need for olecranon osteotomy.

Surgical technique (Fig. 1): In the olecranon osteotomy approach group; the elbow joint is approached posteriorly. After protection of the ulnar nerve an inverted V-shaped Chevron osteotomy is performed 2 cm distal to the olecranon tip to access the joint articular surface. Before that olecranon is drilled and tapped for the future screw. After fracture components reduction, transient fixation may be provided with K wires then definitive fixation achieved with plates in orthogonal configuration. The site of the olecranon osteotomy fixed with a tension band or cancellous screw. In triceps lifting approach the elbow joint is also approached posteriorly, but without any olecranon osteotomy. Articular surface is visualized utilizing the planes medial and lateral to the triceps and elevation of the triceps from posterior aspect of the humerus.

Postoperatively, long arm splint is applied. Sutures were removed at the end of the second week. A rehabilitation program was continued consisting of passive progressive gentle range of motion. Patients were given indomethacin 75 mg/day for heterotopic ossification for 6 weeks and 3 doses of prophylactic antibiotic therapy (cefazolin 40 mg/kg/day). Functional evaluation of the patients is carried out with quick DASH (Disabilities of Arm, Shoulder & Hand) scores at the final 1 year follow up. Other complications including surgical site infections, mal-union and non-union of the fracture were compared for both groups.

Analysis of the data was performed using SPSS 20 (Statistical Package for Social Sciences) software. Frequencies and proportion, means and standard deviation were calculated to describe data. Proportions of fracture outcome were compared between two treatment groups using chi square test whereas means were compared using student t-test. *P* < 0.05 was considered statistically significant.

## 3 Results

**Fig. 2:**
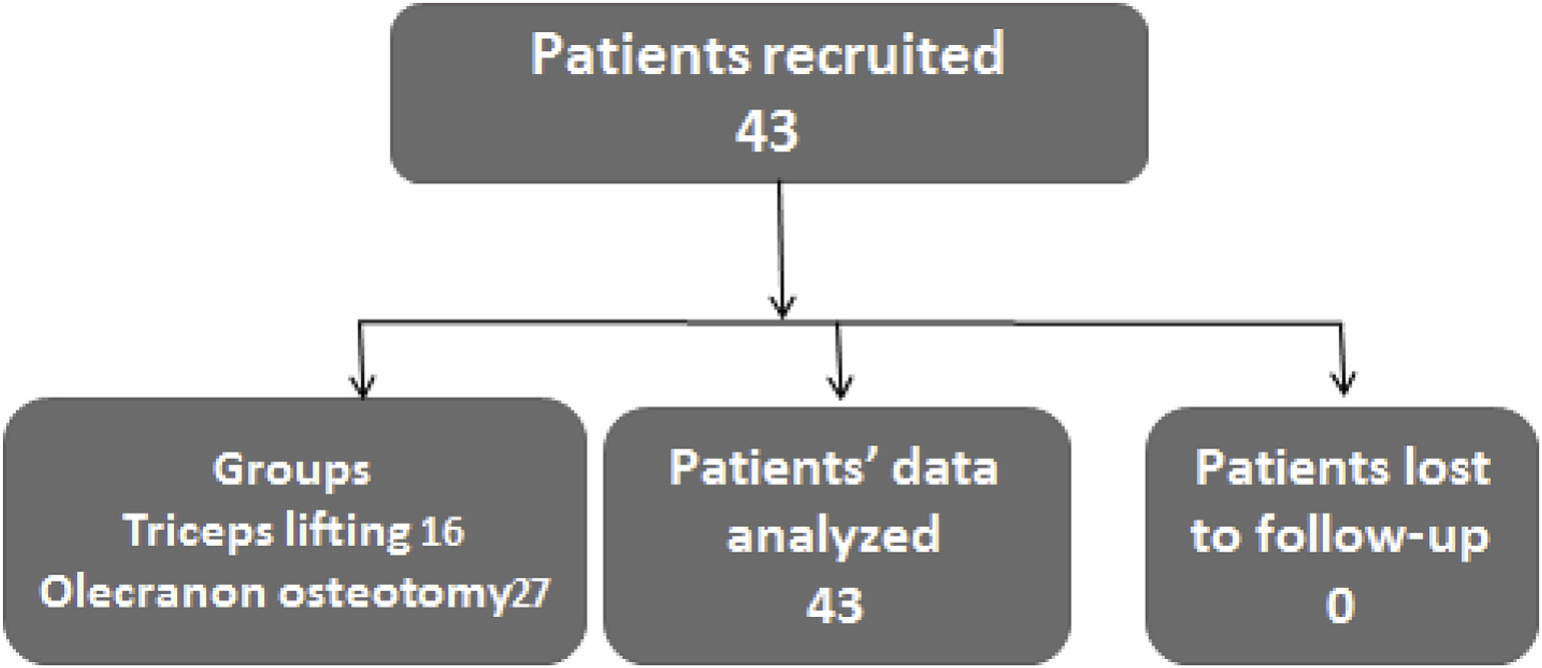
Patents' participation status.

**Fig. 3:**
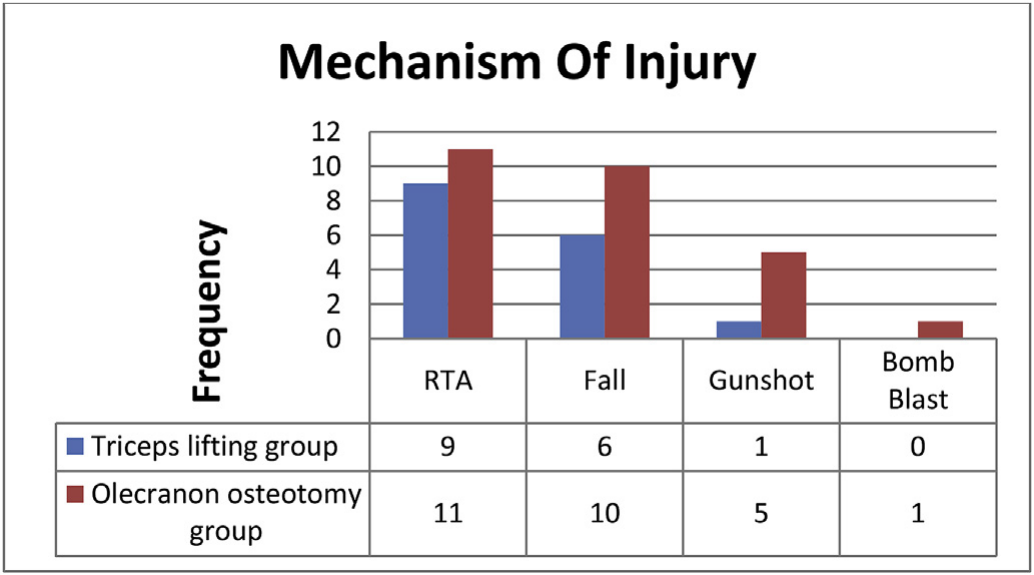
Mechanism of injury in both groups.

We recruited 43 patients who were eligible for the study (Fig. 2). Out of which 16 were treated with triceps lifting approach including 8 males and 8 females and 27 with olecranon osteotomy comprising 23 males and 4 females Table 1. The baseline characteristics of both groups were comparable. Fracture type as per Riseborough Radin Classification in triceps lifting group includes type III in 12 patients and Type IV in 4 patients, whereas in olecranon osteotomy group 13 patients categorized to type III and 14 to Type IV. Mechanism of injury in triceps lifting approach was RTA 9 Fall 6 gunshot 1 and in olecranon osteotomy 11 RTA 10 Fall 5 gunshots and 1 blast was found (Fig. 3). 7 patients in each group were found to have multiple comorbids (DM, HTN, IHD, CKD, and Osteoporosis). In triceps lifting approach mean age was 48.5 years, mean blood loss during surgery was 200.7 ml, mean duration of surgery was 3.3 h and mean quick dash score was 26.8 points while in olecranon osteotomy group mean age 41.7 years, mean blood loss during surgery 226 ml, mean duration of surgery 3.9 h and mean quick dash score 24.9 points was present Table 2. Mean flexion contracture was 8° in both groups with mean arc of motion 8–120° in olecranon osteotomy and mean arc of motion 5–130° in triceps lifting group.

**Table 1:** Demographics (n = 43).

Variable	Triceps lifting approach 16(37%)	Olecranon osteotomy approach 27(63%)
**Gender**		
Male	8 (50%)	23 (85%)
Female	8 (50%)	4 (15%)
**Age (mean years)**	48.5	41.7
**Comorbids**^a^	7 (44%)	7 (26%)
**Mechanism of injury**		
Road Traffic Accident	9(56%)	10 (37%)
Fall	6(38%)	10 (37%)
Gunshot	1(6%)	5 (19%)
Bomb blast	0	1 (4%)
**Riseborough Radin Classification**		
Type III	12(75%)	13 (48%)
Type IV	4(25%)	14 (52%)

a More than 2 comorbids including (Diabetes Mellitus, Hypertension, Ischemic Heart Disease, Chronic Kidney disease and osteoporosis).

**Table 2:** Surgical outcome (n = 43).

Variable	Triceps lifting approach	Olecranon osteotomy approach
Blood loss	200.7 ml	226ml
Surgery duration	3.3 h	3.9 hours
Mean Quick DASH score^a^	26.8	24.9
Range of motion^a^	5–130°	8-120°
Postoperative complications		
Superficial SSI^b^	1 (6%)	0
Delayed union of osteotomy site	Not applicable	2 (7%)

a Quick DASH score and Range Of Motion at 1 year follow-up.

b Managed successfully with oral antibiotics.

The difference between the mean quick DASH score for both groups was not statistically significant (p = 0.52). Quick DASH score for males of Group 1 was significantly lower than for males of Group 2 (6.4 Vs 33.15 with p value < 0.01). Associated complications were comparable for both groups but 2 of the patients in Group 2 suffered delayed union of the osteotomy whereas 1 patient in triceps lifting group suffered superficial surgical site infection managed non-operatively with oral antibiotics.

## 4 Discussion

Both surgical approaches in our study were comparable in terms of functional outcome and radiological union. Both methods provided reliable reproducible results, though complications were lower in triceps sparing approach, but our sample size was not sufficient to demonstrate a statistically significant conclusion. There are multiple techniques reported to approach the distal humerus posteriorly, however, no proved superior to other in terms of functional outcome and less number of complications [9]. Olecranon osteotomy provided excellent exposure and avoiding problems such as intramuscular nerve injuries [10]. Then again, osteotomies, as elsewhere in the body, can be complicated with delayed union, nonunion and a hardware which is being prominent. Proponents of triceps lifting approach find it comfortable in approaching the joint without osteotomy thus avoiding additional fixation.

Olecranon osteotomy approach requires more time for healing of osteotomy in addition to primary fracture, causes prolong recovery with risk of nonunion/malunion at osteotomy site. Patients with the triceps-lifting approach had early mobilization, greater range of motion and short recovery period. Zhang et al. also compared the two approaches in terms of functional outcome after fixation of complex intraarticular distal humerus fractures and reported better outcomes with triceps sparing as compared to olecranon osteotomy [7]. Whereas Chen et al. performed stratified analysis in terms of age while comparing these surgical exposure and identified that elderly patients who underwent the triceps-sparing approach tended to have unsatisfactory functional outcomes and lower MEPS scores [8].

Our study showed comparable functional outcomes with either the two surgical approaches. Both of these approaches can be used effectively for fixation of complex intra-articular distal humerus fractures.

## 5 Conclusion

This study demonstrated no significant differences between both groups in terms of fracture healing, nonunion at the fracture site and functional outcomes although the proportion of complications was also lower with triceps lifting approach at 1 year follow-up. Therefore, we concluded that triceps lifting approach can be used equally efficiently for exposure of these complex distal humerus injuries with no comprise in visibility of articular fragments. However prospective randomized control trial on large volume of patients preferably a multi-centre study may help to prove the reproducibility of our findings.

### 5.1 Strengths

The study design with comparison group to assess the functional outcome between these two surgical approaches. All patients were operated by only one surgeon who is experienced in this procedure and that reduces the bias of learning curve if multiple surgeons were involved, particularly the inter-variability which has impact on precision of the result. We were lucky to analyze all operated patients without missing data, specially related to exposure or outcome, or loss to follow-up which was long enough to derive these conclusions.

### 5.2 Limitations

Among main limitations is the retrospective design of the study and reliability on the medical records. The sample size of our study was relatively small to derive strong associations. All cases were operated by the same surgeon and that could increase the bias in the study, particularly selection bias as he would prefer one approach over the other based on fracture complexity. We only reported the functional outcomes at 1 year without analyzing the short-term outcomes which could be different between both groups. Moreover no documentation or notes were there to evaluate whether the approach was changed during surgery than the planned one or not. Further research, including long term follow up and RCTs with clinical and radiological outcomes will help establish strong conclusions and may show a significant superiority of one approach to the other.

## Ethical Approval

Ethical Approval was obtained before start of study and it was given by out university Ethical Review Committee ERC of Aga Khan University Hospital. Reference number: 3799 ERC-11-dec-15.

## Sources of funding

None.

## Author contribution

**Mohammad Atif:** Study design, data collection, analysis first drat and final approval.

**Obada Hasan:** Study design, Data management, writing the manuscript and final approval.

**Yasir Mohib:** Revising manuscript and final approval for publication.

**Rizwan Haroon Rashid:** Data and results analysis, writing the manuscript and final approval.

**Pervaiz Hashmi:** Supervision, Protocol development and approval, manuscript final approval.

## Conflicts of interest

The authors declare that there are no conflicts of interest in this study.

## Research Registration number

ClinicalTrils.gov.

ID# NCT03833414.

## Guarantor

Mohammad Atif.

Yasir Mohib.

Rizwan Haroon Rashid.

## Provenance and peer review

Not commissioned externally peer reviewed.
